# Real-Time Precise Prediction Dispersion Turning Point of Optical Microfiber Coupler Biosensor with Ultra-High Sensitivity and Wide Linear Dynamic Range

**DOI:** 10.3390/bios15040241

**Published:** 2025-04-10

**Authors:** Haiyang Yu, Yue Wang, Yang Xu, Wenchao Zhou, Yihui Wu

**Affiliations:** 1 Changchun Institute of Optics, Fine Mechanics and Physics, Chinese Academy of Sciences, Changchun 130033, China; yuhaiyang20@mails.ucas.ac.cn (H.Y.); wangyue@ciomp.ac.cn (Y.W.); xuyang@ciomp.ac.cn (Y.X.); 2 University of Chinese Academy of Sciences, Beijing 100049, China; 3 State Key Laboratory of Advanced Manufacturing for Optical Systems, Changchun 130033, China; 4 GD Changguang Zhongke Bio Co., Ltd., Foshan 528200, China

**Keywords:** dispersion turning point, optical microfiber coupler biosensor, mode interference, ultra-high sensitivity, linear dynamic range

## Abstract

Optical microfiber biosensors demonstrate exceptionally ultra-high sensitivity at the dispersion turning point (DTP). However, the DTP is highly susceptible to variations in dimensional and external environmental factors, and the spectral response is mismatched from preparation in air to application in a liquid environment, making the DTP difficult to control effectively. In this work, we propose a method that bridges the relationship between the interference spectra of air and aqueous environments. By counting the interference peaks in air, we can accurately predict the DTP position in liquids. Meanwhile, it provides a new balance between sensitivity and wide linear dynamic range, achieving wide dynamic range detection across various concentrations. The optical microfiber coupler (OMC) is fabricated using the hydrogen–oxygen flame melting tapering method. In addition, the concentration, temperature, and solvent used for the sensor’s biofunctional layer are optimized. Finally, in refractive index sensing, a maximum sensitivity of 1.17 × 105 ± 0.038 × 105 nm/RIU is achieved. For biosensing, a wide dynamic range detection of cardiac troponin I (cTnI) is realized at concentrations of 12–48 ng/mL, 120–480 pg/mL, and 120–480 fg/mL.

## 1. Introduction

Biosensors have been rapidly evolving and widely employed in the fields of ecological monitoring [1,2], food safety [3,4], chemical analysis [5], and clinical medicine [6,7]. However, biosensors have an inherent trade-off between sensitivity and linear dynamic range. Generally, pursuing higher sensitivity comes at the expense of the dynamic linear range [8]. For example, the multimode interferometer offers ultra-sensitive RI detection at 10,675.9 nm/RIU, but the measurement range of the sensor is only 1.4484–1.4513 [9]. In contrast, although the SMS fiber-based planar multimode waveguide is limited to measure the RI with a low sensitivity of 151.29 nm/RIU, a large dynamic range of 1.345–1.405 can be obtained [10]. These mutual constraints have always been a problem that restricts the development and application of biosensors.

Therefore, optical microfiber biosensors [11,12,13] have become a central focus for researchers and developers due to their short response times, high sensitivity, real-time detection, and resistance to electromagnetic interference, demonstrating significant potential in dynamic biomolecular binding monitoring and molecular diagnostics [14]. Notably, recent advancements in microfiber sensors with dispersion turning points (DTPs) open up new possibilities in balancing sensitivity and linear dynamic range [15]. To date, microfiber sensing schemes have been proposed and investigated, examples including microfiber couplers (OMCs) [16,17], microfiber Sagnac interferometers [18,19], and zigzag-shaped tapered optical microfibers (Z-OMFs) [20]. Near the DTP, the sensors can be exploited to achieve ultra-high sensitivities, meanwhile, we found the sensitivity for the same working wavelength varied with the distance from different DTPs whose range can extend from thousands to tens of thousands or even higher. This offers an opportunity to simultaneously achieve high sensitivity and a wide dynamic range.

But, to meet the DTP requirements, the diameters of these sensors are generally below 5 μm [21], and the surfaces require a low roughness to prevent mode energy loss. The sensors are typically fabricated using methods such as the flame heating cone pulling method [22,23,24], chemical etching [21,25], laser processing [26,27], etc. Chemical etching may introduce defects on the optical fiber surface, while laser processing might fail to meet the diameter requirements. Thus, we employed the hydroxide flame melting tapering method [28], a type of flame heating cone pulling method, for the preparation of OMCs. However, in practical biosensing applications, the OMC is typically surrounded by a liquid environment. The significant refractive index (RI) difference between air and liquid leads to changes in the mode interference state. Air and aqueous environments exhibit distinct spectral properties, so accurately predicting the location of the DTP in the liquid in the preparation process is difficult. Additionally, the quality of the biofunctional layer [29,30], including the uniformity and thickness, directly impacts the interaction between biomolecules and the evanescent field, which in turn affects the sensitivity and detection precision of the sensor.

Here, we propose a subtle method for accurately predicting the DTP and improve the constraint relationship between the dynamic range and sensitivity of the OMC sensor, ensuring high sensitivity while expanding the dynamic range. We first studied the critical role of the waist diameter and surrounding refractive index (SRI) in regulating the DTP. Then we established the relationship between the interference spectra in air and the waist diameter of OMC and calculated the position of the DTP at different refractive indices. Our study indicates that the position of the DTP can be characterized and controlled using the interference spectrum in air. More importantly, the relative distance between the operating wavelength and the DTP determines the wavelength shift. By real-time monitoring of the number of interference peaks, the sensitivity of the operating wavelength can be effectively determined. Different sensitivities of the OMC sensor correspond to detection concentrations at varying magnitudes, thereby achieving a balance between the sensor’s high sensitivity and wide dynamic range. In addition, we also optimized the reaction conditions and process parameters to create a uniform and stable modified film layer. Finally, we experimentally verified the feasibility of the method and achieved wide dynamic range sensing for the measured concentration.

## 2. Theoretical Simulation and Numerical Analysis

### 2.1. Operation Principle

**Figure 1:**
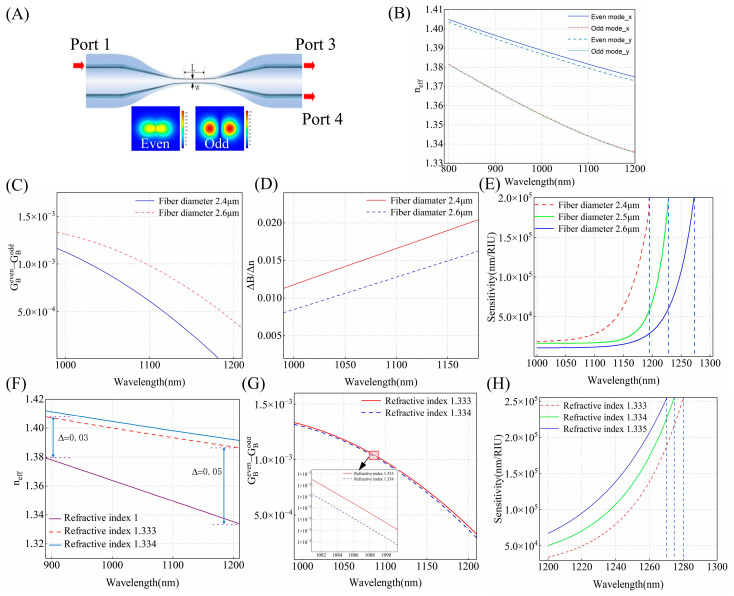
(**A**) Schematic diagram of the microfiber coupler sensor. (**B**) Calculated effective RIs of the guided even and odd modes in *x*-polarization and *y*-polarization. (**C**) The ΔG curves within the wavelength range of 1000–1200 nm for waist diameters of 2.4 μm and 2.6 μm. (**D**) The $\frac{\Delta B}{\Delta n}$ curves within the wavelength range of 1000–1200 nm for waist diameters of 2.4 μm and 2.6 μm. (**E**) The sensitivity curves within the wavelength range of 1000–1300 nm for refractive indices of 1.333 with waist diameters of 2.4 μm, 2.5 μm, 2.6 μm. (**F**) The effective refractive index of the even mode in *x*-polarization for refractive indices of 1, 1.333, and 1.334 within the wavelength range of 900–1200 nm. (**G**) The ΔG curves within the wavelength range of 1000–1200 nm for refractive indices of 1.333, 1.334. (**H**) The sensitivity curves within the wavelength range of 1200–1300 nm for a waist diameter of 2.6 μm with refractive indices of 1.333, 1.334, and 1.335.

Figure 1A shows the schematic structure of a microfiber coupler (OMC), which is composed of two transition regions and a uniform waist region. When the optical signal enters the down-taper region, higher-order modes are excited due to structural changes in the fiber. Near the waist region, the mode field distribution produces superposition. In-phase modes are superimposed as even modes, while out-of-phase modes are superimposed as odd modes. Coupling between these two modes occurs when they propagate along the waist. Due to the high birefringence of the OMC, the modes of *x*-direction and *y*-direction polarization evolve as they propagate along the waist. Consequently, the output power can be expressed as [31]:

(1) $$P_{3}=P_{1x}{cos}^{2}(\frac{\phi_{x}}{2})+P_{1y}{cos}^{2}(\frac{\phi_{y}}{2})$$

(2) $$P_{4}=P_{1x}{sin}^{2}(\frac{\phi_{x}}{2})+P_{1y}{sin}^{2}(\frac{\phi_{y}}{2})$$

where $P_{1x}$, $P_{1y}$ represent the input optical power at the incident port in the *x*- and *y*-polarization directions, respectively, $P_{3}$, $P_{4}$ represent the total output power at port 3 and port 4, respectively, $\phi_{x\;}$ and $\phi_{y}$ represent the phase differences between the two modes in the *x*- and *y*-polarization directions, respectively.

We can express the phase term as [31]:

(3) $$\phi_{x}=\frac{2\pi L(n_{even}^{x}-n_{odd}^{x})}{\lambda }$$

(4) $$\phi_{y}=\frac{2\pi L(n_{even}^{y}-n_{odd}^{y})}{\lambda }$$

where $n_{even}^{x}$, $n_{even}^{y}$, $n_{odd}^{x}$, $n_{odd}^{y}$ are the effective RIs of the even mode and odd mode in *x*-polarization and *y*-polarization, respectively. λ denotes the wavelength and L is the effective coupling length.

When the odd and even modes propagate through the waist, a small change in the external environment alters the interference state between the modes, resulting in a wavelength shift. By taking a small variation with respect to the surrounding RI (SRI), the RI sensitivity of the Nth dip sensitivity can be deduced as [31]:

(5) $$S=\frac{\partial \lambda_{N}}{\partial n}=\frac{\lambda_{N}}{G_{B}^{even}-G_{B}^{odd}}\frac{\partial (B_{even}-B_{odd})}{\partial n}$$

where $G_{B}^{even}=B_{i}-\lambda_{N}\partial B_{i}/\partial \lambda$ represents the group birefringence of the even mode or the odd mode, $B_{even}=n_{even}^{x}-n_{even}^{y}$, and $B_{odd}=n_{odd}^{x}-n_{odd}^{y}$, where *i* denotes even or odd mode.

### 2.2. Numerical Analysis

The position of the DTP is dependent on the diameter of the taper waist and the refractive index of the external environment. Consequently, we numerically calculated the effective refractive indices of the odd and even modes in the OMC to analyze how the waist diameter and SRI affect the DTP. The RI distribution of silicon dioxide is determined using the Sellmeier-type dispersion formula. The waist region was set to 12 mm based on the experimental parameters. Figure 1B displays the effective RIs of the even and odd modes in both polarizations in the wavelength range of 800–1200 nm for an OMC with a waist width of 3 μm. The odd and even modes exhibit similar trends, therefore, the effective refractive index of the even mode is analyzed.

#### 2.2.1. Fiber Diameter

In this section, we investigate how the diameter of the OMC influences the DTP and sensitivity. From Equation (4), the sensitivity is determined by three terms: the wavelength $\lambda_{N}$, $\frac{\Delta B}{\Delta n}=\frac{\partial (B_{even}-B_{odd})}{\partial n}\;$, and the difference between the group birefringence of the even mode and the odd mode $\Delta G=G_{B}^{even}-G_{B}^{odd}$. When the group birefringence difference is zero, the DTP condition is satisfied, resulting in a significant increase in sensitivity, which may even approach infinity. For different diameters, the calculated ΔG between even mode and odd mode for an OMC as a function of wavelength is shown in Figure 1C. The SRI is set to be 1.333, and the waist diameter of the OMC is 2.4 μm and 2.6 μm, respectively. It is evident that, for a specific operation wavelength, ΔG increases with the widening of the waist diameter, and this difference becomes more pronounced as the wavelength becomes longer. The reason is the birefringence of the even mode is more significantly affected by the waist diameter compared to that of the odd mode, and $∆B_{even}\;$ is greater than $∆B_{odd}$. The variation of ΔG determines the interference state of the modes, with smaller ΔG indicating that the phases of the even and odd modes are better matched, leading to higher coupling efficiency, thus exhibiting the characteristic of enhanced sensitivity. In Figure 1D, $\frac{\Delta B}{\Delta n}$ exhibits an opposite trend to ΔG. So, according to the sensitivity formula, narrower-diameter OMCs can achieve higher sensitivity at the same wavelength. We calculated the RI sensitivities of the OMC RI sensors as a function of fiber diameter for different operation wavelengths. From Figure 1E, it can be observed that, when the SRI is 1.333, as the wavelength approaches the DTP, the sensitivity increases sharply toward +∞. As the OMC diameter decreases from 2.6 μm to 2.4 μm, the DTP gradually exhibits a blue shift, moving from 1280 nm to 1190 nm.

#### 2.2.2. Surrounding Refractive Index

Figure 1F displays the effective refractive index of even mode when the SRI is 1.333 and 1. The results clearly show that, for the same fiber diameter, the effective refractive index of SRI is 1.333, which is higher than that of SRI at 1, and as the wavelength varies from 900 to 1200 nm, the difference in effective refractive index gradually rises from 0.03 to 0.05. The increase in SRI makes light propagation more closely approach the ideal total internal reflection conditions and, at the same time, a decrease is observed in ΔG. Figure 1G illustrates that, as the SRI changes from 1.333 to 1.334, ΔG gradually declines. In Figure 1H, the sensitivity curves exhibit a similar trend with increasing wavelength across different SRIs, as the SRI changes from 1.333 to 1.335. At adjacent refractive indices, $\frac{\Delta B}{\Delta n}$ remains constant and, as ΔG gradually decreases, the DTP gradually shifts to a shorter wavelength, moving from 1280 nm to 1270 nm.

#### 2.2.3. Regulation of the DTP

**Figure 2:**
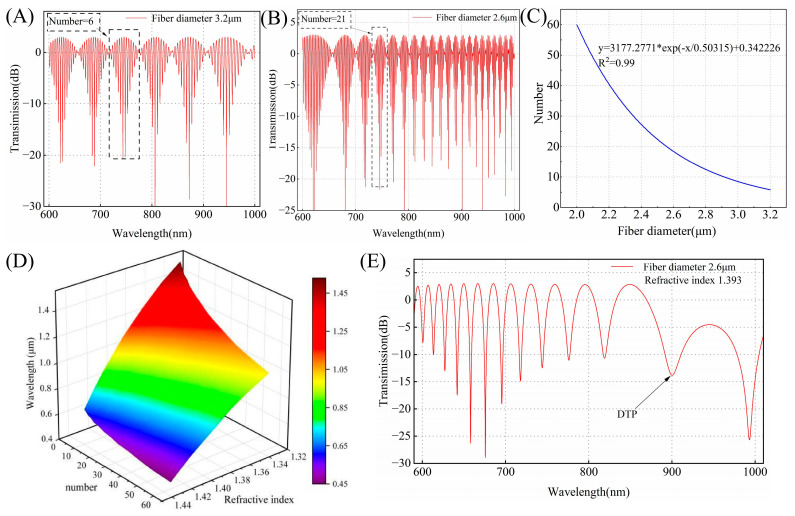
(**A**) The simulated transmission spectrum of the OMC with a waist diameter of 3.2 μm in air. (**B**) The simulated transmission spectrum of the OMC with a waist diameter of 2.6 μm in air. (**C**) The relationship curve between the number of interference peaks and the waist diameter for the OMC in air. (**D**) The three-dimensional surface plot showing the relationship between SRI, the number of interference peaks, and the wavelength position of the DTP. (**E**) The simulated transmission spectrum at the DTP for SRI = 1.393 with a waist diameter of 2.6 μm.

The numerical analysis above indicates that the diameter and external refractive index are determined, and the DTP position is fixed. Thus, it is essential to characterize the diameter during the fabrication process and integrate it with the refractive index of the solution. We calculate the transmission spectra at port 3 for a given effective refractive index (Figure 2A,B). The waist diameters are 2.6 μm and 3.2 μm, respectively. We found the interference fringes in air grow in number with a decrease in the waist diameter. The period of the interference peaks is modulated by the waist diameter. The relationship between the number of characteristic peaks in air and the fiber waist diameter can be established. As depicted in Figure 2C, the number of interference peaks exhibits exponential growth as the optical fiber diameter decreases. This indicates that the optical fiber waist diameter can be determined through the interference spectrum in air. Then, with the diameter fixed, the position of the DTP is calculated for different SRIs. Figure 2D presents a 3D surface plot illustrating how the DTP varies with the SRI and the number of characteristic peaks in air. The number of interference peaks ranges from 6 to 60, and the refractive index varies from 1.333 to 1.343. The accurate prediction of the DTP can be achieved by using the interference spectrum in air as a bridge. For example, when the number of interference peaks in air is 21, we simulate the interference spectra for OMC with a waist diameter of 2.6 μm at an SRI of 1.393. As shown in Figure 2E, the DTP is located at approximately 900 nm. Compared to air, the spectral response shows a longer period. In the 600–1000 nm wavelength range, only a single characteristic peak is observed, and the adjacent peaks or dips gradually broaden as the wavelength increases, with the broadening near the DTP reaching maximum. This demonstrates that the position of the DTP can be adjusted with nanometer-level precision during the preparation process. When the fiber diameter ranges from 3.2 μm to 2.8 μm, the diameter change for each characteristic peak is approximately 66.6 nm per phase difference, and the DTP shift is about 26 nm under an SRI of 1.333. In contrast, when the fiber diameter ranges from 2.2 μm to 2 μm, the diameter change for each characteristic peak is approximately 10.2 nm per phase difference, and the DTP shift is about 4.6 nm under the same SRI. Additionally, the relative distance between the operating wavelength and DTP determines the spectral shift response. For different concentrations of the analyte, an OMC sensor with an appropriate number of interference peaks can be selected to enable sensing with a wide dynamic linear range. These simulation results provide helpful guidelines for the fabrication and sensing of the OMCs.

## 3. Results and Discussion

### 3.1. Materials and Methods

#### 3.1.1. Materials

A single-mode optical fiber with core and cladding diameters of 8.3 µm and 125 µm was purchased from Corning Inc. (New York, NY, USA). Hydrochloric acid (HCL, 37%), acetic acid, methanol, (3-aminopropyl)triethoxysilane (APTES), and glutaric dialdehyde (50%) were purchased from Aladdin Co., Ltd. (Shanghai, China). Phosphate buffered saline (PBS, pH 7.4) solution was purchased from Sigma-Aldrich (St. Louis, MO, USA). Cardiac troponin I antigens and monoclonal cTnI antibodies were supplied from Bioss (Beijing, China). All the chemical reagents were of analytical grade. All solutions were prepared using deionized water. A spectrometer was used as the detector and the data were acquired by a laptop. To eliminate the influence of temperature, the biosensing experiments were carried out in a clean room environment and a temperature of 27 ± 0.2 °C was maintained throughout the experiments.

#### 3.1.2. Experimental Setup

**Figure 3:**
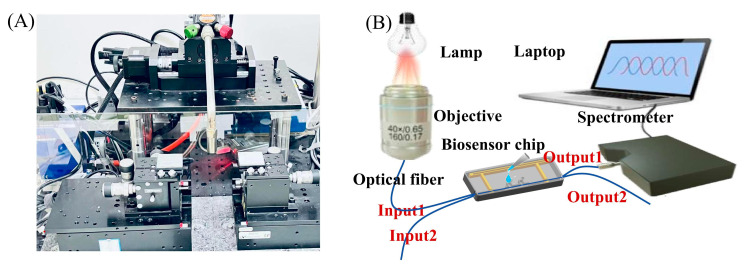
(**A**) System diagram of the OMC fabrication apparatus. (**B**) Illustration of the experimental setup.

The OMC fabrication system is shown in Figure 3A, while the experimental setup is depicted in Figure 3B. The OMCs were prepared by the hydrogen–oxygen flame melting tapering method [28]. The OMC was fixed in a fluid cell chip for the delivery of sample solutions (the chip structure is supplemented in Figures S1 and S2). A halogen lamp was used as the light source, and the light was launched into the fiber using a microscope objective with a numerical aperture of 0.65. A spectrometer was used as the detector, and the data were acquired by a laptop. The wavelength range of the spectrometer is 600−980 nm. The functional layer was modified as follows: first, the surface of the optical fiber was treated with a 1:1 mixture of hydrochloric acid and methanol for 10 min to remove organic contaminants and activate the surface hydroxyl groups. Subsequently, the surface was rinsed thoroughly with deionized water to remove excess solution. The sensor was then dried in a 70 °C drying oven for 10 min to eliminate surface moisture. Next, a 1% APTES solution was applied to the sensing chip to form an APTES monolayer on the sensor surface. The sensor was then washed with acetic acid solution to remove physically adsorbed APTES molecules and heated again at 70 °C to promote cross-linking of the APTES molecules and enhance the stability of the membrane layer. A 1% glutaraldehyde solution was added, followed by the addition of cTnI antibody (10 µg/mL) after 30 min of reaction. The reaction was allowed to proceed for 1–2 h to immobilize the antibody on the surface of the optical fiber through the interaction of carboxyl and aldehyde groups. The optical fiber surface was then thoroughly cleaned with PBS solution to complete the biomodification process. Finally, the OMC is ready for cTnT molecule detection. During the surface modification and biomarker detection process, we inject the samples into the sensor chip and suck out the liquids using a pipette. The volume for each injection is 200 μL.

### 3.2. Biofunctional Layer and Antibody Concentration

#### 3.2.1. Biofunctional Layer

To ensure the reproducibility and consistency of the OMC sensor, we conducted an analysis of the biofunctional layer on its surface. Silane coupling is a widely used surface modification technique that increases the stability and uniformity of the modified layer and facilitates the formation of a highly uniform monomolecular layer on the sensor surface. By precisely controlling and optimizing reaction conditions such as ambient temperature, solvent type, and reagent concentration, a highly reproducible and stable silane layer can be achieved.

**Figure 4:**
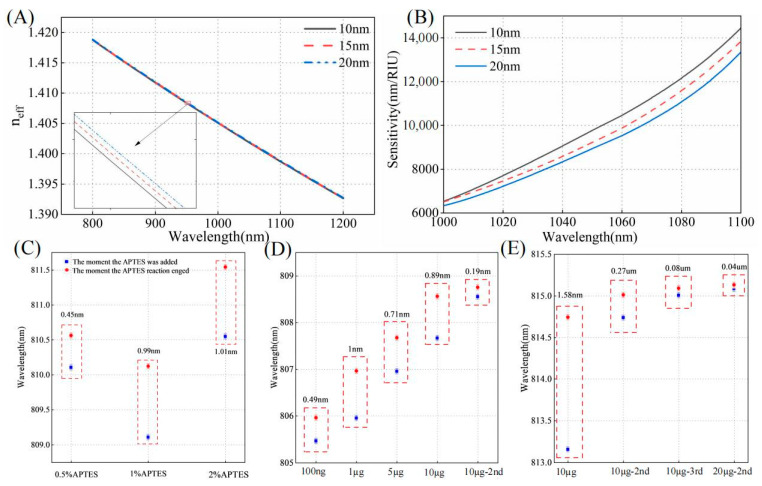
(**A**) The functional layer thicknesses of 10 nm, 15 nm, and 20 nm are evaluated in relation to the effective refractive index of *x*-polarization even mode. (**B**) The sensitivity curves are analyzed for functional layer thicknesses of 10 nm, 15 nm, and 20 nm within the wavelength range of 1000–1100 nm. (**C**) The wavelength shift near 810 nm is analyzed for APTES concentrations of 0.5%, 1%, and 2%. (**D**) The wavelength shift is evaluated for different antibody concentrations immobilized on OMC. (**E**) The wavelength shifts are analyzed for the sequential addition of 10 μg/mL antibodies three times and the subsequent addition of 20 μg/mL antibodies.

The thickness and density of the silane layer under different process conditions significantly influence biodetection performance. As illustrated in Figure 4A,B, simulations of the effective refractive index and sensitivity were conducted for functional layer thicknesses of 10 nm, 15 nm, and 20 nm, respectively. The effective refractive index of the mode gradually increases with the thickness of the functional layer. As the film layer becomes thicker, the detection sensitivity decreases at the same wavelength for a given fiber diameter and SRI. Controlling a thinner mode layer is more advantageous for biosensing. The thickness is closely related to factors such as concentration and solvent; therefore, they are analyzed. The choice of solvent determines the number of hydrolyzed groups in the APTES molecule during hydrolysis. Dissolving APTES in methanol or acetic acid results in a surface closer to a monolayer compared to dissolution in ethanol [32]. Additionally, physically adsorbed APTES molecules can be removed more efficiently through rinsing with acetic acid. Where acetic acid is used as the solvent, the concentration of APTES was analyzed.

**Figure 5:**
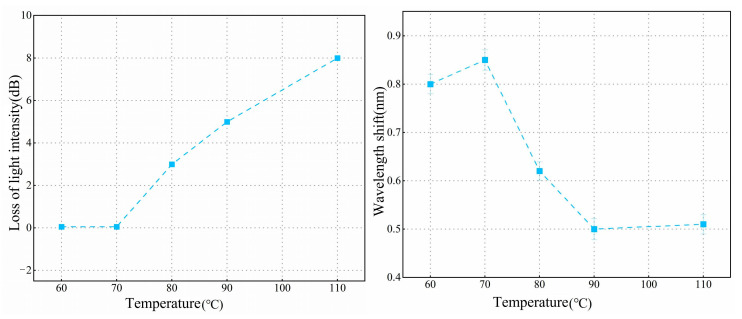
Light intensity loss and wavelength shift of OMCs at different temperatures.

As shown in Figure 4C, at a concentration of 0.5%, the immobilization efficiency and quality of the antibody are both relatively low within the same time frame. Compared to the condition without the addition of antibodies, the transmission spectrum shows a red shift of only 0.45 nm. At a 2% concentration of APTES, the spectral shift differed by only 0.02 nm compared to the 1% concentration, indicating that antibody fixation was nearly saturated at the 1% concentration. Moreover, a 1% concentration tends to form a thinner functional layer. Therefore, a 1% concentration was selected to achieve a more uniform and consistent film layer. To investigate the effect of temperature on silane coupling, we selected temperatures of 60 °C, 70 °C, 80 °C, 90 °C, and 110 °C for experimental testing. The experimental results are presented in Figure 5. At 70 °C, APTES molecules can be better cross-linked, forming a stable silylation layer while preventing any optical fiber morphology changes that might occur at higher temperatures. At this temperature, the OMC light intensity remains almost unchanged, and the antibody immobilization effect is optimal, meeting our requirements for surface modification of the fiber. To ensure the accuracy and reliability of the experimental data, we repeated the experiments three times for each temperature condition and calculated the standard deviation of both light intensity loss and wavelength shift. The minimum standard deviation of light intensity loss was 0.0696 dB, and the minimum standard deviation of the wavelength shift was 0.019 nm. Therefore, we selected 70 °C as the optimal temperature for drying and curing during the silane coupling process.

#### 3.2.2. Antibody Concentration

The bioassay results are closely related to the number of antibodies immobilized on the functional layer. Therefore, we investigated the immobilization of cTnI antibodies at different concentrations on the fiber surface. After functionalizing the optical fiber surface, cTnI antibodies at various concentrations were added sequentially, and the wavelength shift of the spectra was observed. Initially, 100 ng/mL of cTnI antibody was immobilized on the sensor surface and until the reaction reaches equilibrium. Subsequently, cTnI antibodies with concentrations of 1 µg/mL, 5 µg/mL, 10 µg/mL, and 10 µg/mL (second addition of 10 µg/mL) were sequentially added, and the results are shown in Figure 4D. The addition of higher-concentration antibodies still results in a wavelength shift, with the shift amounting to 2.79 nm. This indicates that, even after the reaction with 100 ng/mL antibodies, a significant number of unoccupied binding sites remain on the surface. When 10 µg/mL of antibody was added three consecutive times, the wavelength shift was only 0.08 nm (Figure 4E). When the 10 µg/mL antibody reaction reached equilibrium and 20 µg/mL of antibody was added twice in succession, the wavelength shift was only 0.04 nm, indicating that the binding sites on the surface of the optical fiber were nearly saturated at the 10 µg/mL concentration. Therefore, we ultimately chose to immobilize the antibody at a concentration of 10 µg/mL on the sensor surface.

### 3.3. SRI Sensing and the Detection of cTnI in PBS Buffer

#### 3.3.1. SRI Sensing

**Figure 6:**
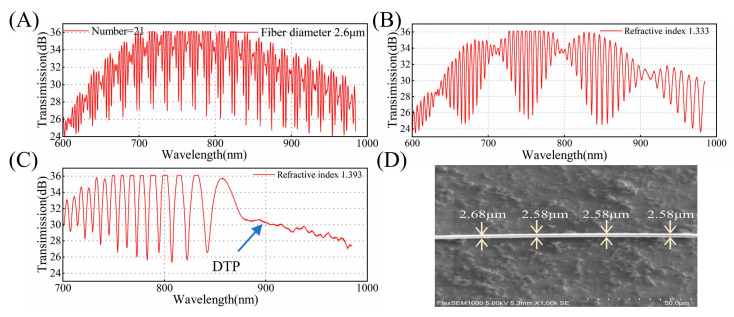
(**A**) The experimental transmission spectrum of the OMC with a waist diameter of 2.6 μm in air. (**B**) The experimental transmission spectrum of the OMC with a waist diameter of 2.6 μm in water. (**C**) The experimental transmission spectrum at the DTP for SRI = 1.393 with a waist diameter of 2.6 μm. (**D**) A scanning electron microscopy (SEM) image of the OMC.

To experimentally verify the theoretical results and demonstrate the feasibility of the proposed method, we fabricated the OMC using the hydrogen–oxygen flame melting tapering method and analyzed its spectral property. The transmission spectra of the same OMC in air or water are shown in Figure 6A,B. The couplers exhibit fewer periodic oscillations and greater spectral broadening in environments with larger refractive indices, which agrees well with the simulated spectra in Figure 2. Figure 6C shows the spectral response with DTP. As the wavelength approaches the DTP, the broadening of adjacent peaks or troughs gradually increases, reaching the maximum at the DTP. Figure 6D is the scanning electron microscopy (SEM) image of the OMC. At a characteristic peak number of 21 in air, the DTP occurs at 900 nm in an aqueous glycerol solution with a refractive index of 1.393 when the fiber diameter is 2.58 μm. When measuring the diameter using SEM, measurement errors may occur due to factors such as charge accumulation. This result shows excellent agreement with the simulation results.

To analyze the sensitivity of different interference peaks, we prepared three OMCs for refractive index sensing detection. The preparation process was conducted with a stretching speed of 0.04 mm/s, a hydrogen flow rate of 130 sccm, a waist length of 12 mm, and a fixation angle of 15° to ensure the fabrication of OMC sensors with high consistency. OMCs with different numbers of interferometric spectra were tested near an SRI of 1.3333, which is typical for biochemical analyses of aqueous solutions. The SRI gradually increased from 1.3333 to 1.3337, with a step 0.0001. The solution’s refractive index (RI) was measured using an Abbe refractometer operating in the visible range. Although measurements in the visible and near-infrared (NIR) ranges may differ slightly, the RI values obtained from the Abbe refractometer serve as a reference for evaluating the performance of the OMC sensor.

**Figure 7:**
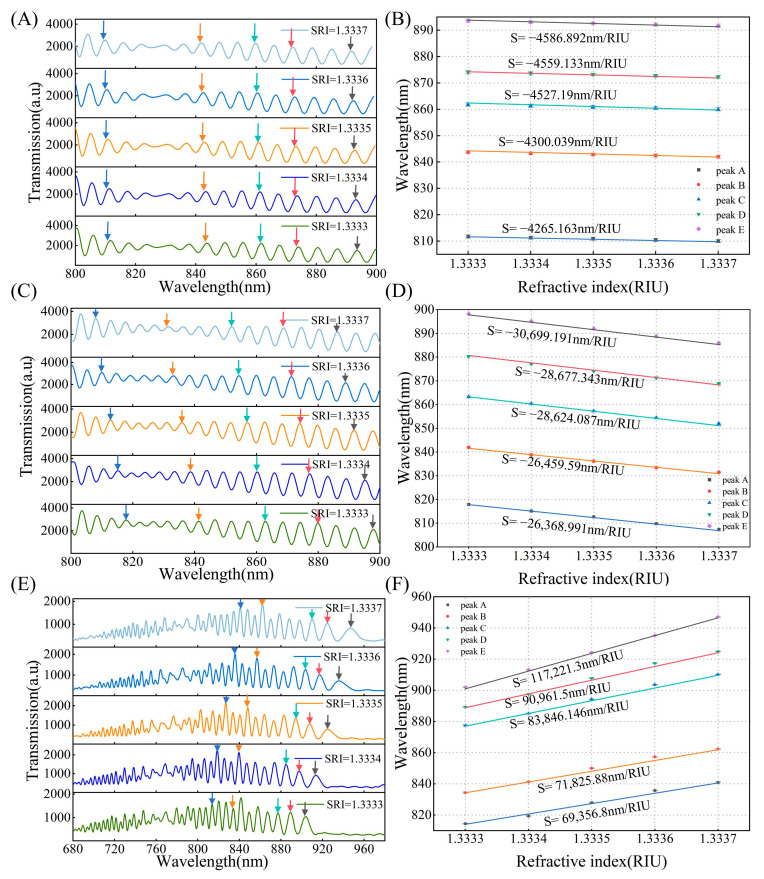
(**A**) The number of interference peaks in air is 20, transmission spectral response to SRI range from 1.3333 to 1.3337, different colored arrows are to guide the shift of interference peaks as ambient RI increases, as follows. (**B**) Wavelength shifts of interference peaks versus SRI, peaks A, B, C, D, and E correspond to different colored arrows, as follows (**C**) The number of interference peaks in air is 30, transmission spectral response to SRI range from 1.3333 to 1.3337. (**D**) Wavelength shifts of interference peaks versus SRI. (**E**) The number of interference peaks in air is 65, transmission spectral response to SRI range from 1.3333 to 1.3337. (**F**) Wavelength shifts of interference peaks versus SRI.

In the experiment, we gradually added the prepared aqueous glycerol solution to the cuvette in small increments. To ensure the accuracy of the measurement, we replaced the solution in the cuvette with an identical solution of the same refractive index (RI) six times until the spectrum stabilized before conducting the measurement. The variation in the number of characteristic peaks directly affects the DTP position, and the greater the number of interference peaks and their proximity to the DTP, the higher the sensitivity at the same wavelength. We tested the sensors in the wavelength range of 600–980 nm, with 20, 30, and 65 characteristic peaks, respectively. As shown in Figure 2D, under an SRI condition of 1.333, the DTP is located at approximately 1285 nm, 1150 nm, and 975 nm, respectively. Figure 7A,C,E illustrate the relationship between the transmission spectrum and SRI under different numbers of interference peaks. The transmission spectra with 20 and 30 interference peaks in air exhibit a blue shift of the interference peaks as the refractive index increases. For the transmission spectrum with 65 interference peaks, the DTP appears at 975 nm, the interference peaks shift towards the DTP. The DTP gradually shifts to shorter wavelengths, which are in good accordance with our previous numerical results. Figure 7B,D,F show the sensitivity corresponding to different peak values. The RI sensitivity increased significantly as the inclination angle approached the DTP. When there are 20 interference peaks, the sensitivity ranges from 4000 nm/RIU to 5000 nm/RIU, while for 30 interference peaks, it ranges from 20,000 nm/RIU to 30,000 nm/RIU, ultimately reaching an ultra-high sensitivity of 1.17 × 10^5^ ± 0.038 × 10^5^ nm/RIU near the DTP. The wavelength is approximately 840 nm, and the standard deviations of the three fibers are less than 0.2 nm (*n* = 6). It is obvious that different numbers of characteristic peaks correspond to varying sensitivities. Therefore, in the application of such a sensor, we have evaluated the concentrations to be measured first and then chose the appropriate number of interference peaks. This approach helps resolve the conflict between sensitivity and detection range to some extent while improving the utility of OMC sensors to achieve accurate detection over a broad dynamic range.

#### 3.3.2. Sensitivity and the Dynamic Linear Range

**Figure 8:**
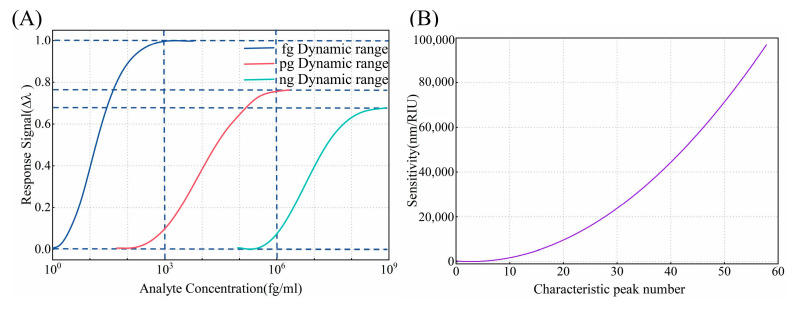
(**A**) The sensitivity versus the number of characteristic peaks in air. (**B**) The schematic diagram of the dynamic range for different concentrations.

Sensitivity is a critical performance characteristic in biosensor technology, defining a biosensor’s response capability to detect the smallest measurable change in analyte concentration. The dynamic linear range in biosensors refers to the range of target concentrations in which the sensor exhibits a linear signal response between the minimum and maximum detectable signals [8]. Figure 8A demonstrates the sensor’s response behavior across different concentration ranges, where the horizontal axis is presented on a logarithmic scale and the vertical axis represents the signal response intensity, i.e., the wavelength shift. The signal response is not always directly proportional to the concentration, especially at very low or high levels. Thus, selecting an appropriate sensitivity for different target concentrations to achieve a wide dynamic linear range in sensing will greatly facilitate the practical application of the sensor. For example, the detection concentration range for AFP typically falls within the ng/mL range, which does not require an extremely low detection limit but instead demands a broader dynamic detection range. In contrast, cTnI, as a biomarker for myocardial infarction, is present in very low concentrations in the blood, requiring enhanced sensitivity for reliable detection. In this study, we use cTnI as a proof of concept to validate the proposed method. As shown in Figure 8B, there is an exponential relationship between the number of characteristic peaks and sensitivity. During the preparation process, we can observe the number of interference peaks to assess the sensor’s sensitivity, matching the sensitivity with the linear dynamic range to achieve more precise sensing.

#### 3.3.3. The Detection of cTnI in PBS Buffer

Acute myocardial infarction (AMI) is one of the leading causes of death globally, and early diagnosis is critical for patient prognosis [33]. Cardiac troponin I (cTnI) is widely recognized as a key biomarker for AMI, providing accurate indications of cardiac injury, and plays an essential role in the diagnosis of heart diseases. Numerous studies have demonstrated that, in the early stages of acute myocardial infarction (AMI), cTnI levels change significantly and increase rapidly, making it a highly sensitive biomarker for the early detection of AMI. cTnI has a typical range of 0.1 to 0.04 ng/mL, but readings of high-sensitivity cardiac troponin (hs-cTn) are less than 14 ng/L [34,35]. In recent years, various detection techniques have been widely applied to the measurement of cardiac troponin I (cTnI), including colorimetry, fluorescence, paramagnetism, and electrochemical methods [36]. S.M. Seo and colleagues [37] employed a fluorescence detection method for cTnI, achieving a detection limit as low as 0.002 ng/mL and a detection range from 0 to 12.5 ng/mL. This method demonstrates high sensitivity, but its detection range is relatively narrow. On the other hand, S.M. Khoshfetrat and collaborators [38] utilized an electrochemical detection method for cTnI, with a detection limit of 0.01 ng/mL and a broader detection range from 0.01 ng/mL to 250 ng/mL. Although the detection range is expanded, the sensitivity is somewhat reduced. Therefore, achieving an optimal balance between high sensitivity and a wide dynamic range in practical applications is crucial.

**Figure 9:**
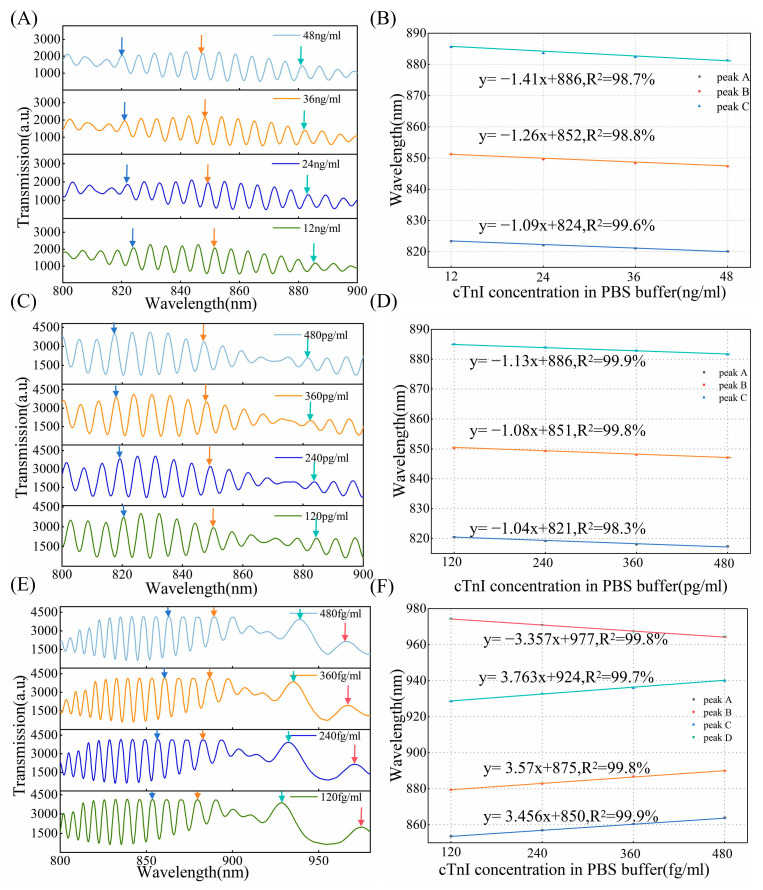
(**A**) The number of interference peaks in air is 20, transmission spectral response of different cTnI concentrations, different colored arrows are to guide the shift of interference peaks as cTnI antigen concentration increases, as follows. (**B**) Linear response of cTnI binding to the anti-cTnI immobilized on the surface of the OMC at a concentration in the range of 12–48 ng/mL (*n* = 3), peaks A, B, and C, correspond to different colored arrows, as follows. (**C**) The number of interference peaks in air is 30, transmission spectral response of different cTnI concentrations. (**D**) Linear response of cTnI binding to the anti-cTnI immobilized on the surface of the OMC at a concentration in the range of 120–480 pg/mL (*n* = 3). (**E**) The number of interference peaks in air is 65, transmission spectral response of different cTnI concentrations. (**F**) Linear response of cTnI binding to the anti-cTnI immobilized on the surface of the OMC at a concentration in the range of 120–480 fg/mL (*n* = 3).

To demonstrate the effectiveness of the method, detection of cTnI in PBS buffer was carried out. The cTnI antigen–antibody binding was performed by injecting 200 μL of cTnI antigen solution with concentration ranges of 12–48 ng/mL, 120–480 pg/mL, and 120–480 fg/mL into the chip, allowing the reaction to proceed for 10 min at room temperature. We use OMCs with 20, 30, and 65 characteristic peaks to perform detection, respectively. The transmission spectra of the biosensor for concentrations ranging from 12 ng/mL to 48 ng/mL are shown in Figure 9A. By comparing the spectra of different cTnI concentrations, it is shown that the interference wavelengths gradually undergo a blue shift as the antigen concentration increases, with the shift being more pronounced at longer wavelengths. This is caused by the binding of the cTnI molecules which can lead to small increases in the SRI. And longer wavelengths are closer to the DTP, therefore exhibiting higher sensitivity. Figure 9B shows that the sensor demonstrates a good linear response in the range of 12–48 ng/mL and, near the wavelength of 820 nm (R^2^ = 0.996), the standard deviation was 0.1 nm (*n* = 3). Figure 9C,D illustrate the transmission spectra and the sensitivity variation trend in the cTnI antigen concentration range of 120–480 pg/mL. At lower concentrations of 120–480 pg/mL, the shifts of the peak can be clearly distinguished and have the same behavior across concentrations ranging from 12 to 48 ng/mL. Additionally, the consistency of the interferometric wavelength offset is high under the same concentration difference condition. The measurement was repeated times using three fibers with good consistency. Around the wavelength of 820 nm (R^2^ = 0.983), the standard deviation was 0.2 nm (*n* = 3). By leveraging the ultra-high sensitivity characteristics of the DTP, we detected cTnI antigens in the range of 120–480 fg/mL. Figure 9E,F present the transmission spectra and linear response for 120–480 fg/mL. It is clear that the DTP is observed near 955 nm. Due to the modification of the functional layer and the difference in refractive index, the position of the DTP is slightly shifted, similar to the SRI sensing characteristics at the DTP. The interference peaks on the left side of the DTP undergo a red shift, with a significant change occurring as they approach the DTP, and the interference peaks on the right side of the DTP exhibit a blue shift. The interferometric peak shifts show a good linear response to the concentration (R^2^ = 0.999). The slope of the fitting curve for the sensor with the DTP is much larger than that without the DTP, even though it detects lower concentrations, indicating the ultra-high-sensitivity characteristics of the DTP. At the wavelength of 880 nm, the standard deviation was 0.25 nm (*n* = 3). The results show that predicting the DTP position and assessing sensitivity through the number of interference peaks in air, which can more accurately focus on concentration detection, enable wide dynamic range sensing, thereby enhancing applicability in both chemical and biological sensing areas.

A high-sensitivity OMC sensor with a wide dynamic range for biosensing is achieved by precisely controlling the DTP position and optimizing the surface biofunctionalization process. When the fiber preparation conditions are consistent, the preparation repeatability is approximately 80%, which is attributed to errors during the fixation of the fiber. This method can also be implemented in other optical microfiber sensors with DTP structures that establish functional relationships to modulate the DTP. It is worth noting that our study focused solely on OMCs with a waist diameter of 2–3.2 μm. However, the functional relationship between the number of interference peaks and the diameter is also applicable when the waist diameter is below 2 μm. Additionally, we exemplified OMC sensors with 20, 30, and 65 interference peaks based on their sensitivity range and performed detection, but this did not fully cover all concentration ranges. Altering the number of interference peaks, other concentrations of cTnI can also be detected. A more suitable OMC sensor can be selected according to the concentration indicators of various biomolecules. The detection of cTnT is presented solely as a proof of concept to demonstrate the proposed sensor’s biosensing capability and evaluate the effectiveness of the dynamic range methods used for biosensing applications.

## 4. Conclusions

In conclusion, we have achieved precise prediction of DTP in solution, and the sensitivity and linear dynamic range of the OMC at various DTP positions have been investigated, resulting in an enhanced balance between the two factors. The effective refractive index of the mode for different diameters and refractive indices has been numerically investigated, and the results indicate that the DTPs can be tuned for a number of interference peaks from 6 to 60 with nanometer accuracy. As a proof of concept, RI sensitivities of −4586.892 nm/RIU, −30,699.191 nm/RIU, and 117,221.3 nm/RIU have been achieved. Meanwhile, we have achieved linear detection of cTnI across 12–48 ng/mL, 120–480 pg/mL, and 120–480 fg/mL concentration ranges and experimental results confirmed the wide dynamic range of ng/mL–fg/mL and high-sensitivity sensing. Additionally, we investigated the effects of solvents, concentrations, and heating temperatures on the preparation of the silane coupling biofunctionalization layer. Through process optimization, we have obtained a biofunctional layer with high uniformity and consistency. This sensing approach offers a generic platform for optical microfiber with a DTP for highly sensitive, wide dynamic range linear detection in the biomedical field.

## Data Availability

Data are contained within the article and Supplementary Materials.

## Supplementary Materials

The following supporting information can be downloaded at: https://www.mdpi.com/article/10.3390/bios15040241/s1, Figure S1: Optical fiber surface electron microscopy diagram and physical picture of the fixed bracket. Figure S2: Sensor chip structure diagram.
