# Supplementary material for: Real-Time Precise Prediction Dispersion Turning Point of Optical Microfiber Coupler Biosensor with Ultra-High Sensitivity and Wide Linear Dynamic Range
Source: Biosensors (Basel). 2025 Apr 10;15(4):241. doi: 10.3390/bios15040241 (PMC12025059; doi:10.3390/bios15040241)
Supplement: Supplementary file 1 [file biosensors-15-00241-s001.zip › biosensors-3517200-supplementary.pdf]

SUPPORTING INFORMATION

Real-Time Precise Prediction Dispersion Turning Point of Optical Microfiber Coupler Biosensor with Ultra-High Sensitivity and Wide Linear Dynamic Range

Haiyang Yu[1,2,3], Yue Wang[1,3], Yang Xu[1,3,4], Wenchao Zhou[1,3,]* and Yihui Wu[1,3,4,]*

1 Changchun Institute of Optics, Fine Mechanics and Physics, Chinese Academy of Sciences, Changchun, Jilin 130033, China; yuhaiyang5285@163.com(H.Y.); wangyue@ciomp.ac.cn(Y.W.); xuyang@ciomp.ac.cn(Y.X.); zhouvc@ciomp.ac.cn(W.Z.); yihuiwu@ciomp.ac.cn(Y.W.)

2 University of Chinese Academy of Sciences, Beijing 100049, China ; yuhaiyang5285@163.com(H.Y.)

3 State Key Laboratory of Advanced Manufacturing for Optical Systems, Changchun, Jilin 130033, China; yuhaiyang5285@163.com(H.Y.); wangyue@ciomp.ac.cn(Y.W.); xuyang@ciomp.ac.cn(Y.X.); zhouvc@ciomp.ac.cn(W.Z.); yihuiwu@ciomp.ac.cn(Y.W.)

4 GD Changguang Zhongke Bio Co., Ltd., Foshan, Guangdong, 528200, China. xuyang@ciomp.ac.cn(Y.X.); yihuiwu@ciomp.ac.cn(Y.W.)

* Correspondence: zhouvc@ciomp.ac.cn(W.Z.); yihuiwu@ciomp.ac.cn(Y.W.)

Preparation of optical fiber couplers.

The OMCs were prepared by the hydrogen-oxygen flame melting tapering method (Ref 1). The optical fiber couplers preparation setup is presented in Figure 3(A) of the manuscript, with the following detailed steps: First, a suitable length of single-mode fiber is selected, and its coating is removed to expose the bare fiber for subsequent processing. The prepared fibers are then fixed onto a fiber holder and wound at a predetermined angle to ensure stable relative positioning during the tapering process. Next, the system control module automatically positions the flame nozzle, aligning it with the heating area and following a preset scanning path with a scanning distance of 1 mm. After igniting the oxyhydrogen flame (with a hydrogen flow rate of 130 sccm), a 2-minute preheating operation is carried out to stabilize the system thermally. Once preheating is completed, the high-precision displacement platform is activated to stretch the fiber along its axial direction (at a speed of 0.04 mm/s), while the flame displacement platform ensures uniform heating of the stretching area. During the

tapering process, an interferometer is used to monitor changes in the interference spectrum in real-time, and the fiber diameter is adjusted based on shifts in the interference peaks. When the desired interference spectrum is achieved, the stretching operation is immediately stopped, completing the fabrication of the micro-nano fiber. Finally, the tapered fiber is precisely aligned with the microfluidic chip, and the fiber is fixed and packaged.

Fixed package and chip structure.

In this study, the fixation of the optical microfiber coupler was achieved using a six-degree-of-freedom displacement platform, ensuring high precision alignment and fixation. Using this platform, the uncoupled ends of the optical microfiber coupler were precisely aligned with the ports of the fixed bracket, and ultraviolet adhesive was applied to secure the coupler, ensuring its stability and reliability.

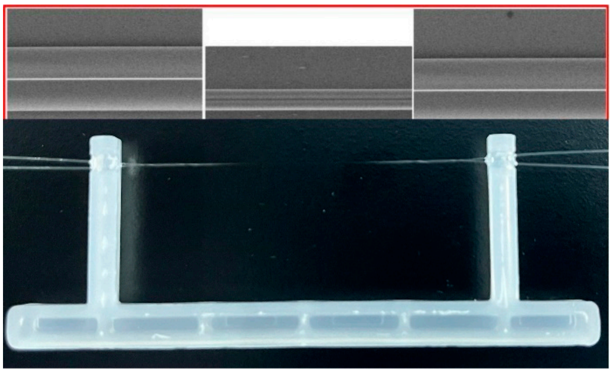

Figure S1. Optical fiber surface electron microscopy diagram and physical picture of the fixed bracket

The fixed bracket is shown in Figure S1. After the ultraviolet adhesive is cured, the OMC is transferred into the pre-designed sensor chip, ensuring a tight and secure integration between the fiber and the sensor chip.

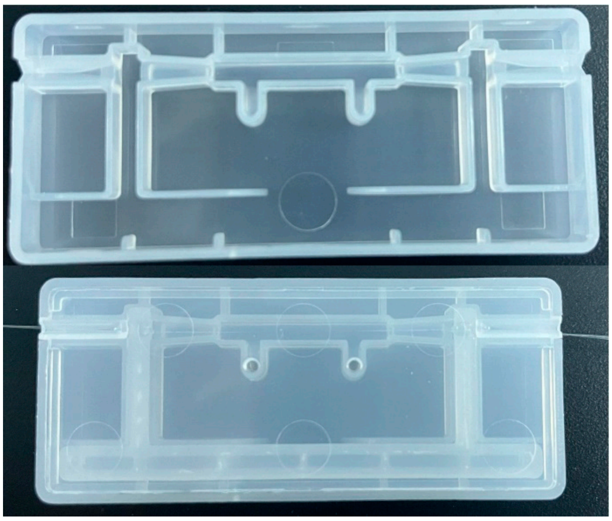

Figure S2. Sensor chip structure diagram

The sensor chip used in this study features an optimized design structure (as shown in Figure S2). The chip is made of polypropylene (PP), a material known for its excellent corrosion resistance. This property effectively prevents the corrosion of the sample chamber by acidic or alkaline solutions, thereby avoiding contamination of the sensor. Additionally, the stability of polypropylene ensures that the chip maintains superior reliability under various experimental conditions. To ensure the stability of the optical fiber sensor during use and prevent damage, the OMC is enclosed with a snap-fit packaging sheet after being placed at the bottom of the reaction chamber. This packaging sheet not only securely holds the coupler in place but also features sample inlet and outlet holes, facilitating the injection and outflow of samples. The design of the packaging sheet ensures that the reaction chamber provides enough space to accommodate sufficient liquid sample, ensuring that the optical fiber is completely surrounded by the liquid and shielded from external interference. To further optimize the performance of the optical fiber sensor, appropriate tapering is incorporated at both ends of the reaction chamber, which not only guarantees the stability of the fiber but also effectively prevents liquid leakage, ensuring the protection of the optical fiber throughout the experiment. In addition, the design of this study also takes into account the need for solution replacement. When a solution change is required, the system allows for easy replacement of the bottom sample chamber, ensuring that the sensor is not affected by the previous solution. This feature enhances the repeatability and accuracy of the experiments. Such a structural design effectively improves the durability and multifunctionality of the sensor, making it adaptable to various experimental environments.

REFERENCES

1. Xu Y, Fang W, Tong L. Real-time control of micro/nanofiber waist diameter with ultrahigh accuracy and precision. Optics Express. 2017; 25(9): 10434-10440.
